# Exendin-4 Pretreated Adipose Derived Stem Cells Are Resistant to Oxidative Stress and Improve Cardiac Performance via Enhanced Adhesion in the Infarcted Heart

**DOI:** 10.1371/journal.pone.0099756

**Published:** 2014-06-10

**Authors:** Jianfeng Liu, Haibin Wang, Yan Wang, Yujing Yin, Liman Wang, Zhiqiang Liu, Junjie Yang, Yundai Chen, Changyong Wang

**Affiliations:** 1 Department of Advanced Interdisciplinary Studies, Institute of Basic Medical Sciences; Tissue Engineering Research Center, Academy of Military Medical Sciences, Beijing, P.R. China; 2 Department of Cardiology, Medical school of Chinese PLA, Chinese PLA General Hospital, Beijing, P.R. China; 3 Department of Geriatric Cardiology, Chinese PLA General Hospital, Beijing, China; 4 Institute of Transfusion Medicine, Academy of Military Medical Sciences, Beijing, P.R China; Georgia Regents University, United States of America

## Abstract

Reactive oxygen species (ROS), which were largely generated after myocardial ischemia, severely impaired the adhesion and survival of transplanted stem cells. In this study, we aimed to determine whether Exendin-4 pretreatment could improve the adhesion and therapeutic efficacy of transplanted adipose derived stem cells (ADSCs) in ischemic myocardium. *In vitro*, H_2_O_2_ was used to provide ROS environments, in which ADSCs pretreated with Exendin-4 were incubated. ADSCs without pretreatment were used as control. Then, cell adhesion and viability were analyzed with time. Compared with control ADSCs, Exendin-4 treatment significantly increased the adhesion of ADSCs in ROS environment, while reduced intracellular ROS and cell injury as determined by dihydroethidium (DHE) staining live/Dead staining, lactate dehydrogenase-release assay and MTT assay. Western Blotting demonstrated that ROS significantly decreased the expression of adhesion-related integrins and integrin-related focal adhesion proteins, which were significantly reversed by Exendin-4 pretreatment and followed by decreases in caspase-3, indicating that Exendin-4 may facilitate cell survival through enhanced adhesion. *In vivo*, myocardial infarction (MI) was induced by the left anterior descending artery ligation in SD rats. Autologous ADSCs with or without Exendin-4 pretreatment were injected into the border area of infarcted hearts, respectively. Multi-techniques were used to assess the beneficial effects after transplantation. Longitudinal bioluminescence imaging and histological staining revealed that Exendin-4 pretreatment enhanced the survival and differentiation of engrafted ADSCs in ischemic myocardium, accompanied with significant benefits in cardiac function, matrix remodeling, and angiogenesis compared with non-pretreated ADSCs 4 weeks post-transplantation. In conclusion, transplantation of Exendin-4 pretreated ADSCs significantly improved cardiac performance and can be an innovative approach in the clinical perspective.

## Introduction

Ischemic heart disease is the major cause of death globally. Although there are several kinds of therapeutic strategies, such as medical, interventional approaches and heart transplantation, the mortality rate of patients with acute myocardial infarction (AMI) is still very high [1]. Over the past years, stem cell-based cardiac reconstruction was emerging as a robust means to repair damaged myocardium and provided novel opportunities for the therapies of AMI.

However, infarcted and peri-infarcted myocardium exhibits a hostile niche, such as largely generated reactive oxygen species (ROS), enhanced inflammatory response and vascular dysfunction. All these factors were detrimental to engrafted stem cells. Studies confirmed that a major number of transplanted cells detached from the impaired myocardium or died within 1 week after injection [2], [3], which was defined as anoikis (a programmed cell death resulting from loss of cell-matrix interactions) [4]. The cell adhesion to the extracellular matrix (ECM) proteins is critical for transplanted cell fate, including proliferation, migration, differentiation and survival [5], [6], [7], [8]. Recent evidences suggested that exposure to exogenous hydrogen peroxide (H_2_O_2_) could increase ROS formation in ADSCs and attenuated cell adhesion, whereas the use of ROS scavengers, such as antioxidant, can reverse the impaired adhesion mediated by integrin [9], [10]. Previously, several approaches have been developed to enhance transplanted cell survival in ischemic myocardium through increasing their adhesive ability, including pretreatment before transplantation and genetically engineering [11], [12], [13]. For example, Laflamme *et al*. developed a multicomponent, pro-survival cocktail that limits cell death after transplantation and enhances function of infarcted hearts. The pro-survival cocktail included Matrigel to prevent anoikis [14]. Cho *et al*. genetically engineered MSCs to overexpress periostin to ultimately increase cell survival and improve the cardiac function of infarcted myocardium after implantation. Genetic modification of MSCs with periostin enhanced cell adhesion, survival rates, and cell adhesion-related signaling *in vitro* [15]. In comparison, the method of pretreatment of stem cells before transplantation was more easily and suitable of clinical application.

Exendin-4 is a native glucagon-like peptide (GLP-1) analogue with insulinotropic property. Recent studies showed that GLP-1 and its analogues provided favorable protection for BM-MSCs, such as stimulation of proliferation and inhibition of apoptosis [16], suggesting that pretreatment of stem cells with Exendin-4 may be promising in stem cell-based therapy. Indeed, it has been reported that the agents exhibited antioxidant properties through reduction of endogenous ROS production in human umbilical vein endothelial cells (HUVECs) [17] and in Goto-Kakizaki (GK) islets [18]. Besides, Exendin-4 treatment has successfully been applied to the protection of oxidative stress induced β-pancreatic cells apoptosis [19]. However, whether Exendin-4 pretreatment could significantly attenuate ROS-induced adhesion impairment in ADSCs and further improve ADSC-based myocardial repair following MI is unknown.

In the present study, we investigated the effect of Exendin-4 on adhesive properties and viability of rats ADSCs *ex vivo*, and further evaluated the therapeutic efficacy of autologous ADSCs pretreated by Exendin-4 in rat model of myocardial infarction.

## Materials and Methods

### Animals

All procedures were in accordance with the *Guide for the Care and Use of Laboratory Animals* published by the US National Institutes of Health (NIH Publication, 8th Edition, 2011) and approved by the Institutional Animal Care and Use Committee (IACUC) of Academy of Military Medical Science. Male Sprague-Dawley (SD) rats were purchased from the Experimental Animal Center, Academy of Military Medical Science (Beijing, PRC). Animals were individually housed in cages (accessible to water and food) with a room temperature of 24±2°C (a normal day/night cycle). Six rats were used for cell culture study and 102 rats were used in the *in vivo* MI model study, among them, 4 died during or immediately after the infarct surgery, and two rats died during the injection surgery but there was no mortality in remaining 96 rats (n = 24/group) till their sacrifice at different time points up to 4 weeks.

### Isolation, characterization and lentiviral labeling of ADSCs

ADSCs were obtained from inguinal subcutaneous adipose tissue of SD rat as previously reported [10],[20]. The immunophenotype of ADSCs, including CD29, CD90, CD45, CD34, and CD31, were analyzed by flow cytometer (BD). The verification of osteogenic and adipogenic differentiation were performed by Alizarin Red staining and Oil Red O staining as described previously [21]. The ADSCs were lentivirally transduced to express both firefly luciferase and monomeric red fluorescent protein (fluc-mRFP) as described previously [10], [22]. The 5% highest mRFP expressing cells were selected by FACScan (BD FACSVantage Diva) and expanded before usage. Passage 2–4 ADSCs were used in this study. Details are described in supplementary material in File S1.

### Cell adhesion assays

Assays for cell adhesion were performed in compliance with previous study with slightly modification [23]. Viable ADSCs were suspended in growth medium. Suspensions of 1×10^5^ cells/mL in 2 mL suspensions (2×10^5^ cells) were then added to each well of a six-well plate and allowed to attach for 30 min, 60 min and 120 min at 37°C under 5% CO_2_ incubator, respectively. In order to evaluate the protection of Exendin-4 on ADSCs against ROS, 30 µM hydrogen peroxide (H_2_O_2_) [10] was used as the ROS source. To quantify ADSCs adhesion, plates were carefully washed three times with PBS, and the four separate fields were counted under a phase-contrast microscope. Each experiment was performed in triplicate wells and repeated at least three times at each time point of detections.

### Measurement of intracellular ROS

To evaluate ROS production by ADSCs, dihydroethidium (DHE) staining was employed according to manufacturer's instruction as previously reported [10]. Briefly, cells were pretreated with interventions and then loaded with DHE (1 mM, Invitrogen) for 10–20 min in dark. After washing twice with PBS, The cells were observed by fluorescence microscopes (Olympus). The intensity of DHE staining was quantified using IPWIN60 software (Media Cybernetics, Inc.). Each experiment was repeated at least three times.

### Measurement of cell viability, lactate dehydrogenase release and Caspase-3 activity

The cells were pretreated with 50 nM Exendin-4 (Sigma) for 24 h in 37°C, 5% CO_2_ incubator. Then, the cells were treated with or without 30 µM H_2_O_2_ for 12 h, cell viability was evaluated by live/dead staining (Gibco) according to manufacturer's instructions. LDH release was measured in the cell supernatants by a commercially available kit (Sigma-Aldrich) according to the manufacturer's instructions. Caspase-3 activity was detected by using a Caspase-3 Activity Assay kit (Cell signaling) according to the manufacturer's instructions.

### Cell proliferation assay

ADSCs were plated in a 96-well plate at a density of 1×10^4^/well. After pretreatment with 50 nM Exendin-4, H_2_O_2_ was given to the cells for 6 h, a total of 5 g/L 3-(4, 5-dimethylthiazolyl-2)-2,5-diphenyltetrazolium bromide (MTT) (Sigma) in medium was added to the cells for 2 h. Then the medium was removed and dimethyl sulphoxide (DMSO) was applied at a volume of 200 µL/well. The absorbance was quantified by spectrophotometry at 490 nm. The experiment was repeated 6 times.

### Quantitative Real-Time Polymerase Chain Reaction (qRT-PCR)

ADSCs that were treated by H_2_O_2_ with or without Exendin-4 were collected. Total RNA was extracted from cells using RNAprep pure cell/Bacteria Kit (TIANGEN) and the instructions provided by the manufacturer. First-strand cDNA was synthesized using Thermo First cDNA Synthesis Kit (Germany) according to the standard procedures. The qPCRs were performed in triplicate with the FastStart Universal SYBR Green Master (ROX; Roche, Mannheim, Germany) and run on the StepOnePLUS system (Applied Biosystems, USA). Primers of β-actin used as an internal standard were: forward (5′-GCTACAGCTTCACCACCACA-3′), reverse (5′-GCCATCTCTTGCTCGAAGTC-3′). Two main genes that mediated cell adhesion, integrin αV and the integrin β1,were analyzed. The primers of integrin αV were: forward (5′-AATGTCAGCCCAGTCGTGTCTTAC-3′), reverse (5′- CCAACGTCTTCTTCAGTCTC-3′). The primers of integrin β1 were: forward (5′- ACTAAAGTGGAAAGCAGGGAGAA-3′), reverse (5′- GAAATAGAACCAGCAGTCATCAAT-3′). Samples were run in duplicate with RNA preparations from three independent experiments. The qRT-PCR was performed with an Applied Biosystems StepOne PLUS.

### Western blotting

Cells were washed with PBS and lysed in Laemmli Sample Buffer (Bio-Rad) and further homogenized with a rotorstator homogenizer. Proteins were isolated and concentrations were determined using the BCATM Protein Assay Kit (Thermo Scientific). 80–120 µg proteins were loaded on a 12–15% sodium dodecyl sulfate-polyacrylamide gel. After electrophoresis, proteins were transferred to a PVDF Western Blotting membrane (Roche). Membrane were blocked with 5% nonfat dried milk (in TBST) for 2 h at room temperature and then incubated overnight at 4°C with Primary antibody (p-FAK, FAK, p-Src, Src, paxillin, vinculin, and talin were purchased from abcam, cleaved-caspase3, caspase3 and β-actin were purchased from Cell Signaling Technology).The membrane was subsequently washed with TBST (5 min×3) and incubated with horseradish peroxidase-conjugated secondary antibodies (Cell Signaling Technology) for 1 h at room temperature. After washing with TBST (5 min×3), bands were detected by enhanced chemiluminescence substrate (Applygen). Band intensities were normalized to its respective internal standard signal intensity. The experiment was repeated 6 times.

### Myocardial infarction model and ADSCs transplantation

Rats (n = 96) were randomly divided into the following four groups in a blind study: (24 rats in each group): (1) sham-operated group (sham), (2) the PBS injection group (PBS), (3) ADSCs-transplanted group (ADSCs), (4) Exendin-4 pretreated ADSCs transplanted group (Ex-ADSCs). AMI was induced in the open chests of male Sprague-Dawley (SD) rats (240–260 g) as described previously[20]. Rats were intraperitoneally anesthetized with sodium pentobarbital (30 mg/kg). Limb-lead electrocardiography was performed sequently. The animals were then incubated and ventilated by a volume-regulated respirator during surgery. After a left lateral thoracotomy and pericardectomy, the left coronary artery was identified and gently ligated with a 6.0 prolene suture at approximately 2–3 mm from its origin between the pulmonary artery conus and the left atrium. Successful AMI was confirmed by the typical ST segment elevation in electrocardiography. Then, 5×10^6^ autologous ADSCs in 100 µL PBS were injected along peri-infarct zone at three injected foci with a 28-gauge needle. 100 µL PBS alone was injected as PBS group. Injections were made at an angle to reduce the chance of the injection into the lumen of the LV. Injections were verified by a slight lightening in the color of the myocardium as the solutions entered the infarcted wall. The sham operation group underwent thoracotomy and cardiac exposure with neither coronary artery ligation (suturing without tying the left anterior descending coronary artery) nor cell transplantation. During the surgical procedures, the adequacy of anaesthesia was monitored using absence of the pedal withdrawal reflex, slow constant breathing, and no response to surgical manipulation. Buprenorphine was administered before and after the procedures (0.05 mg/kg, i.p). All the rats received anti-microbial therapy (penicillin, intramuscular twice daily for 3 days).

### Optical Bioluminescence imaging

The correlation between mRFP/fluc-positive ADSCs and *ex vivo* bioluminescence intensity was confirmed by preparing various numbers of cells (per well) in a 96-well plate. Cardiac bioluminescence imaging was performed on all rats using the Xenogen optical marcroscopic imaging system by a blinded researcher. Rats (n = 12/group) were anaesthetized initially with 3.5% isofluorane and maintained with 1.5–2% isofluorane. The adequacy of anesthesia was monitored by toe pinch. For analgesia, buprenorphine (0.05 mg/kg) was given intraperitoneally. After intraperitoneal injection of the reporter probe D-luciferin (375 mg/kg body weight), anaesthetized rats were imaged for 30 minutes with 2-minute acquisition intervals until peak signal was observed. The same rats were longitudinally subjected to BLI on days 1, 7, 14, 28 after AMI. Bioluminescence density was quantified in units of maximum photos per second per centimeter squared per steradian.

### Echocardiogram and hemodynamics

Echocardiography was performed in all rats at 4 weeks after cell delivery. Rats (n = 16/group) were anaesthetized using 1.5–2.0% isofluorane for function measurement with echocardiogram (14.0 MHz, Sequoia 512; Acuson, Germany). Both two-dimensional and M-mode images were recorded. The Left-ventricular end-diastolic diameter (LVEDD) and left-ventricular end-systolic diameter (LVESD) were measured. LV fractional shortening (FS) and LV ejection fraction (EF) were calculated as follows: FS(%) =  [(LVEDD-LVESD)/LVEDD]×100; EF(%) = [(EDD^3^-ESD^3^)/EDD^3^]×100. All procedures and analyses were performed by an experienced and blinded researcher.

After cardiac function measurement, hemodynamic parameters on arterial and LV were assessed with a 2F conductance catheter introduced into the ascending aorta and the left ventricle through the right carotid artery in a subset of the rats (n = 8/group). For analgesia, buprenorphine (0.05 mg/kg) was given intraperitoneally. The data were acquired after stabilization.

### Histological analysis and Immunofluorescence staining

The animals were euthanized after 1 week of transplantation (n = 4) for myocardial apoptosis determination, 2 weeks (n = 4) for ADSCs engraftment analysis, and after functional measurements (n = 16) with an overdose of sodium pentobarbital in compliance with above guidelines. Hearts were removed, fixed in 4% paraformaldehyde, embedded into O.C.T compound, quickly frozen in −80°C, and then processed for crysectioning (4 µm thickness). Ten sections were prepared at 10 different transversal levels at the site of tissue necrosis, equally distributed from base to apex. Masson's Trichrome were performed in sections to quantify the ratio of collagen fiber. The degrees of collagen fiber accumulation in the infarcted area were evaluated by measuring the percentage of fibrotic region in the LV area, which was calculated using RS Image Pro, version 4.5 (Media Cybernetics, Inc., Trenton, NJ).

To evaluation the engraftment of ADSCs, the sections were stained with an anti-cTnI antibody. Cell engraftment was confirmed by identification of mRFP expression under fluorescent microscopy. The numbers of mRFP^+^ cells and DAPI in each slide were calculated. The data were expressed as the percentage of mRFP^+^/DAPI in 5 slides obtained from 5 frozen sections.

The differentiation of ADSCs *in vivo* was identified by cTnT and α-SMA immunofluorescent staining. Briefly, immunofluorescent staining was performed on 4 µm sections using following primary antibodies: anti-cardiac troponin T 1∶200 (abcam) and anti-α-SMA 1∶200 (sigma). Cryosections were fixed with acetone for 30 min and endogenous peroxide activity was quenched with 3% H_2_O_2_. After blocking with 2% normal goat serum, sections were incubated with the primary antibodies at 4°C overnight. Then, FITC-conjugated IgG were incubated for 2 h at room temperature before observing under laser confocal microscope (FV1000, Olympus). Ten high-magnification fields of each section were chosen randomly. The number of the mRPF-positive cardiomyocytes (cTnT+/mRFP+) was calculated. mRPF-positive cardiomyocytes were defined by cells having clear striation staining pattern of cardiac troponin-T. The rate of mRFP-positive cardiomyocytes was defined as the number of the cTnT+ mRFP cells divided by the number of mRFP cells. The number of the mRPF-positive vascular smooth muscle cells (SMA+/mRFP+) was calculated, too. The rate of mRFP-positive vascular smooth muscle cells was defined as the number of the SMA+ mRFP cells divided by the number of mRFP cells.

Vascular density was determined in the section stained with anti-vWF antibody (1∶200, Sigma), FITC-conjugated IgG (Sigma, 1∶100) were incubated for 1 h at room temperature before observing under an Olympus fluorescent microscopy. The number of microvessels was calculated in five randomly high magnification fields. Microvessels in each section were quantified using the following criteria: a) positive for vessel smooth muscle labeling within peri-infarction region; b) having a visible lumen; and c) having a diameter between 10 and 100 µm. The density of arteriole was expressed as the quantity of microvessels per mm^2^. All of these assays were performed in a blinded manner.

### Determination of myocardial apoptosis

Terminal deoxynucleotidyl transferase dUTP nick end labeling (TUNEL) staining was performed on myocardial frozen sections according to the manufacturer's instructions (MEBSTAIN Apoptosis kit II; Takara). Digital images were acquired at high magnification by using a fluorescent microscopy (Olympus) and the infarct area was manually traced on the blue channel. For each heart sample, 50 random high resolution fields (10 sections at different transversal levels, 5 random fields for each section) were chosen and counted in a blinded manner.

### Statistical analysis

All data are expressed as mean±SD. Statistical analyses were performed with SPSS software (version 17.0). Statistical significance between two groups was determined by Student's *t* test. Results for more than two groups were evaluated by one-way ANOVA with Least-significant Difference (LSD) test. *p*<0.05 was considered as significantly difference.

## Results

### Characterization of ADSCs

Rat ADSCs displayed a spindle shape. After three passages, flow cytometric analysis showed that most adherent cells were positive for mesenchymal stem cell surface marker CD29 and CD90, while they were negative for CD34, CD45 and CD31 (Fig. S1A in File S1.). These results revealed that cultured ADSCs were homogeneous without hematopoietic cells contamination, consistent with previous reports [24]. The multipotency of ADSCs was confirmed by their ability to differentiate into adipocytes, osteoblasts (Fig. S1B, C in File S1.).

### Exendin-4 pretreatment inhibits impaired adhesion of ADSCs and increases cell survival under ROS environment

**Figure 1:**
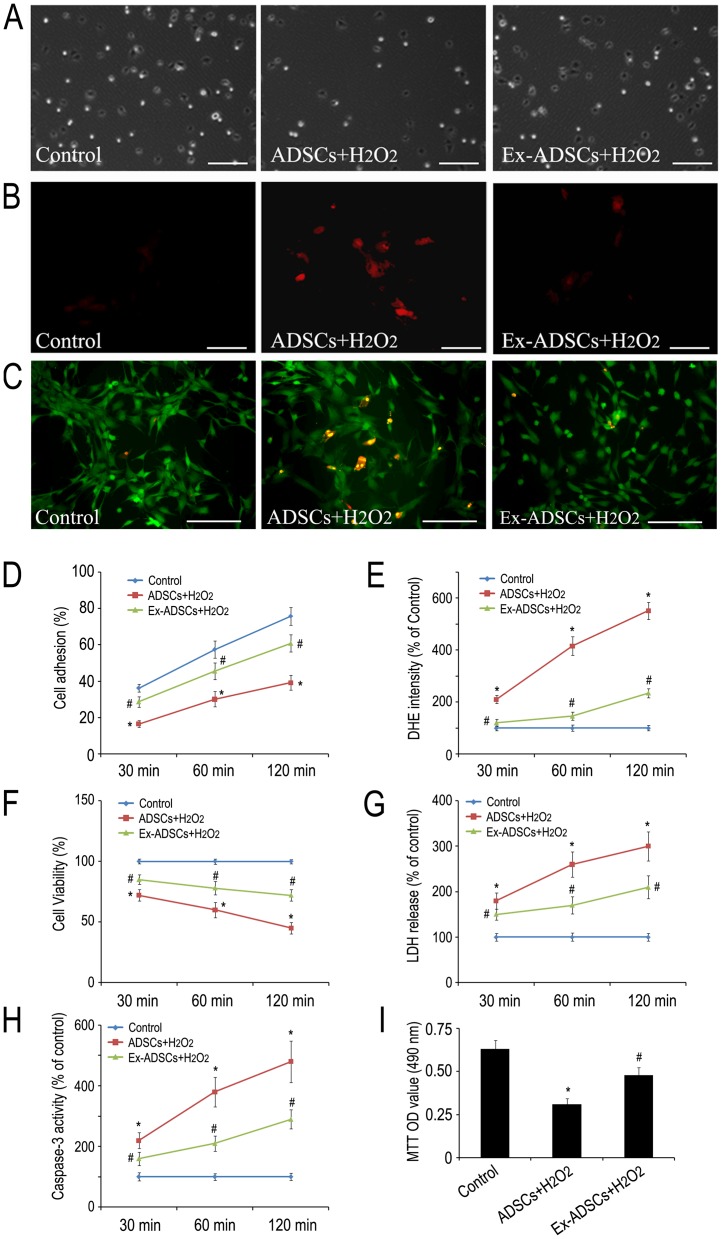
Exendin-4 restores ROS-induced attenuation of ADSCs adhesion, increases their viability and promotes ADSCs proliferation at 30, 60, 120 min after pretreatment. (A) The effect of Exendin-4 on ADSCs adhesion in the presence of H_2_O_2_. Scale bars = 200 µm. (B) The effects of Exendin-4 on scavenging intracellular ROS of ADSCs which were treated with H_2_O_2_ with or without Exendin-4. ROS of ADSCs were detected using DHE reagent. Scale bars = 100 µm. (C) Live/dead staining showed that the effects of Exendin-4 pretreatment on ADSCs viability against H_2_O_2_. Scale bars = 100 µm. (D) Quantification of adhesive ADSCs. (E) Quantification of intracellular ROS. (F) Quantification of viable ADSCs. (G) Quantitative analysis of LDH release in the cell supernatant. (H) Caspase-3 activity determined by using Caspase-3 ELISA kit. (I) MTT assay was performed to analyze the effect of Exendin-4 on viability of ADSCs after H_2_O_2_ injury for 6 h. Statistical differences (*p*<0.05) are indicated from Control group(*) and ADSCs+ H_2_O_2_ group (#).

To investigate the effects of Exendin-4 on the attenuated adhesion and cell survival of ADSCs, we selected the well-established ADSCs/H_2_O_2_ system. Our previous study showed that 30 µM exogenous H_2_O_2_ could significantly inhibit ADSCs adhesion to the culture plate via increased ROS production in ADSCs [10]. We showed here that Exendin-4 pretreatment significantly reverses H_2_O_2_ induced reduction of adhesive ADSCs at 30 min, 60 min and 120 min (Fig. 1A, D; *p*<0.05). DHE staining demonstrated that increased intracellular ROS in ADSCs induced by H_2_O_2_ was partially inhibited by 50 nM Exendin-4 pretreatment from 30 min to 120 min (Fig. 1B, E; *p*<0.05). The cell viability evaluated by live/dead staining suggested that the necrotic rate of ADSCs increased gradually compared to control within 120 min after exposure to H_2_O_2_. This increase in ADSCs necrotic rate can be partially reversed by Exendin-4 pretreatment (Fig. 1C, F; *p*<0.05). Likewise, Exendin-4 pretreatment demonstrated obvious protection of ADSCs against H_2_O_2_ as shown by decreased release of LDH and caspase-3 activity compared to control group (Fig. 1G, H; *p*<0.05). MTT assay showed that treatment of ADSCs with H_2_O_2_ led to significant reduction in cell proliferation, while pretreatment of ADSCs with Exendin-4 showed a significant increase in cell proliferation (Fig. 1I, *p*<0.05). Together, these findings indicated that the impaired ADSC adhesion by ROS was correlated with increased cell apoptosis or decreased cell survival. Exendin-4 could serve as a favorable factor to restore adhesion and improve survival of ADSCs under H_2_O_2_ environments.

### Effects of Exendin-4 on impaired adhesion-related molecules induced by ROS

**Figure 2:**
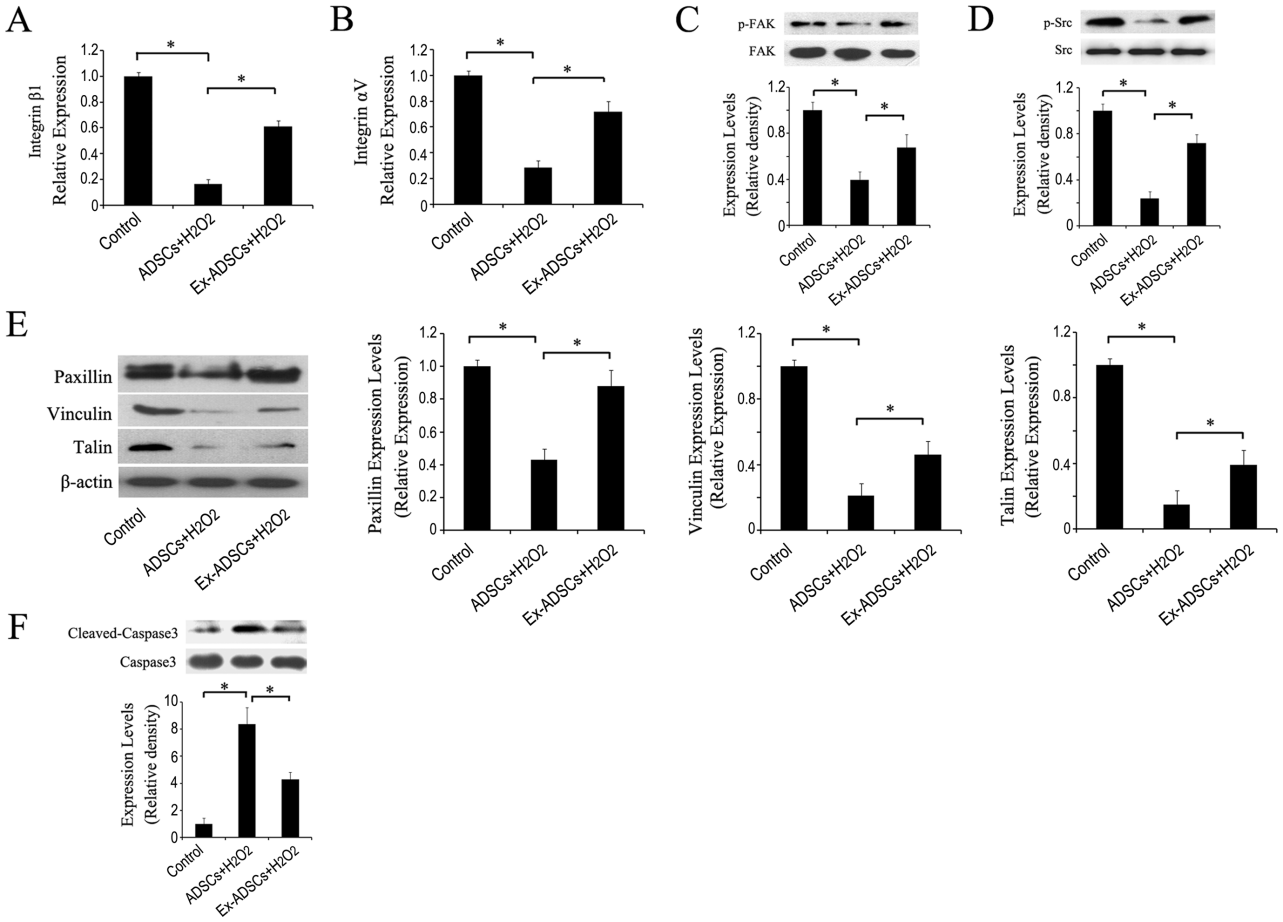
Exendin-4 attenuates integrin-related adhesion protein impairment induced by H_2_O_2_. (A and B) mRNA expression levels of integrin β1 and αV in ADSCs with or without Exendin-4 under H_2_O_2_ injury by qRT-PCR analysis. (C-G) Representative western blotting of p-FAK, p-Src, paxillin, vinculin, talin and caspase-3 expression levels in ADSCs with or without Exendin-4 under H_2_O_2_ injury. ADSCs were pretreated with 50 nM Exendin-4 for 24 h and then treated with or without 30 µM H_2_O_2_ for 12 h. *p<0.05.

To identify the mechanisms of Exendin-4 promoting adhesion of ADSCs, we firstly focused on integrins. It has been found that integrin β1 and αV mediated MSCs adhesion, migration and engraftment in myocardium [25], [26], [27]. The qRT-PCR analysis showed that the mRNA level of integrin β1 and αV in H_2_O_2_ treated cells were significantly down-regulated compared with that in control (*p*<0.05), whereas the reduced mRNA level of both integrins could be partially restored by Exendin-4 pretreatment (Fig. 2A, B). These results indicated that integrin molecules played important roles in Exendin-4 promoted the adhesion of H_2_O_2_-treated ADSCs.

Previous study had reported that cellular focal adhesion-related kinase focal adhesion kinase (FAK) and steroid receptor coactivator (Src) can be activated by integrins at the adhesive stage [28]. In addition, FAK and Src signaling played important roles in the control of adhesion fate [29]. We found that H_2_O_2_ treatment markedly significantly decreased the phosphorylation levels of both kinase (p-FAK and p-Src) compared with that of the control ADSCs. Interestingly, the decreased phosphorylation by H_2_O_2_ was significantly rescued in the presence of Exendin-4 (Fig. 2C, D) (*p*<0.05). These results suggested that integrin-dependent adhesion signaling is involved in the Exendin-4-mediated adhesion in ADSCs. Moreover, the focal adhesion-related proteins, including paxillin, vinculin, and talin, were significantly decreased in exogenous ROS-treated ADSCs but were rescued by Exendin-4 (Fig. 2E) (*p*<0.05). Furthermore, western blotting assay revealed that H_2_O_2_ treatment increased the level of caspase3 dramatically compared to that of control ADSCs, which could be significantly recovered by Exendin-4 pretreatment (Fig. 2F) (*p*<0.05). These data indicated that Exendin-4 was sufficient to enhance cellular survival under oxidative stress conditions through activated adhesion. Together, these findings showed that Exendin-4 facilitated adhesion of ADSCs through the activation of integrin-related survival signaling pathways.

### Exendin-4 promotes the survival and engraftment of transplanted ADSCs

To noninvasively track transplanted cells, we transduced ADSCs with a lentiviral vector expressing fluc-mRFP reporter. FACS sorting analysis indicated that the percentage of mRFP was ∼99% as shown in the representative image Fig. S1D in File S1.. In addition, BLI showed a strong linear correlation between cell number and BLI intensity (Fig. S1E in File S1.), suggesting that the reporter was competent to accurately quantify ADSCs.

**Figure 3:**
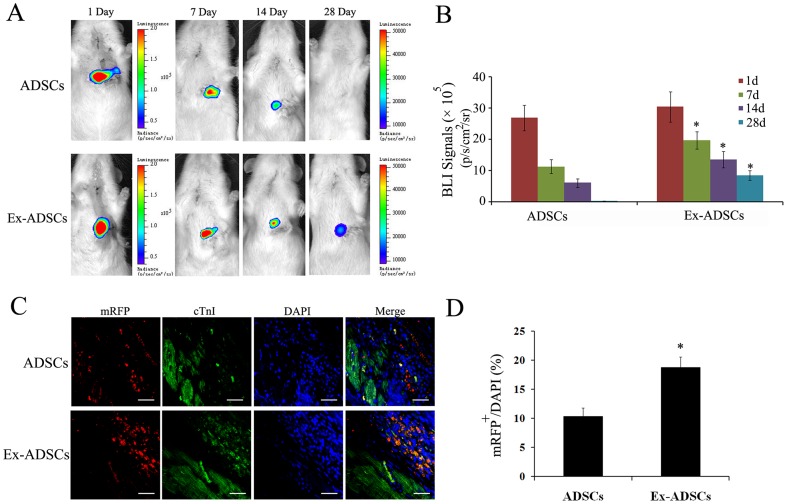
Survival and engraftment of transplanted ADSCs. (A) Representative images of in vivo BLI at days 1, 7, 14 and 28 (n = 12/group). The BLI signal decreased gradually from day 1 to day 28 after cells injection in both groups. But the signals in the Ex-ADSCs group were significantly higher than those in the ADSCs group. (B) Quantitative analysis of serial BLI signal showed a moderate signal was still observed in the Ex-ADSCs group at day 28 after transplantation. (C) Confocal laser microscopic images of ADSCs (fluc-mRFP), cardiomyocytes (cTnI, red florescence) and DAPI (blue fluorescence) at 2 weeks after transplantation (n = 4/group). Scale bars = 50 µm (D) Quantitative analysis of the ratio of fluc and mRFP double-positive cells. *p<0.05.

After transplantation, the injection of ADSCs or Ex-ADSCs led to a strong bioluminescence signal at day 1. During the initial 7–14 day, serial imaging of the same rats confirmed a gradually decreased bioluminescence signal, but the BLI signal intensities of Ex-ADSCs group were constantly stronger than that of ADSCs group (*p*<0.05). At day 28, nearly no signals could be detected in ADSCs group, indicating a great loss of engrafted ADSCs. In comparison, modest signals were still detected in Ex-ADSCs group (Fig. 3A, B).

To further confirm in vivo BLI results for ADSCs survival, cell engraftment was further evaluated by calculating the ratio of mRFP^+^/DAPI cells at 2 weeks after transplantation. The mRFP^+^ cells were more frequently observed in the Ex-ADSCs group (Fig. 3C). The ratio of mRFP^+^/DAPI cells in Ex-ADSCs group was 18.7±1.9%, significantly higher than that in the ADSCs group (10.3±1.5%, *p*<0.05; Fig. 3D). These results suggested that Exendin-4 pretreatment could significantly improve the survival and engraftment of injected ADSCs in ischemic myocardium.

### Transplantation of Ex-ADSCs significantly improves cardiac function

**Figure 4:**
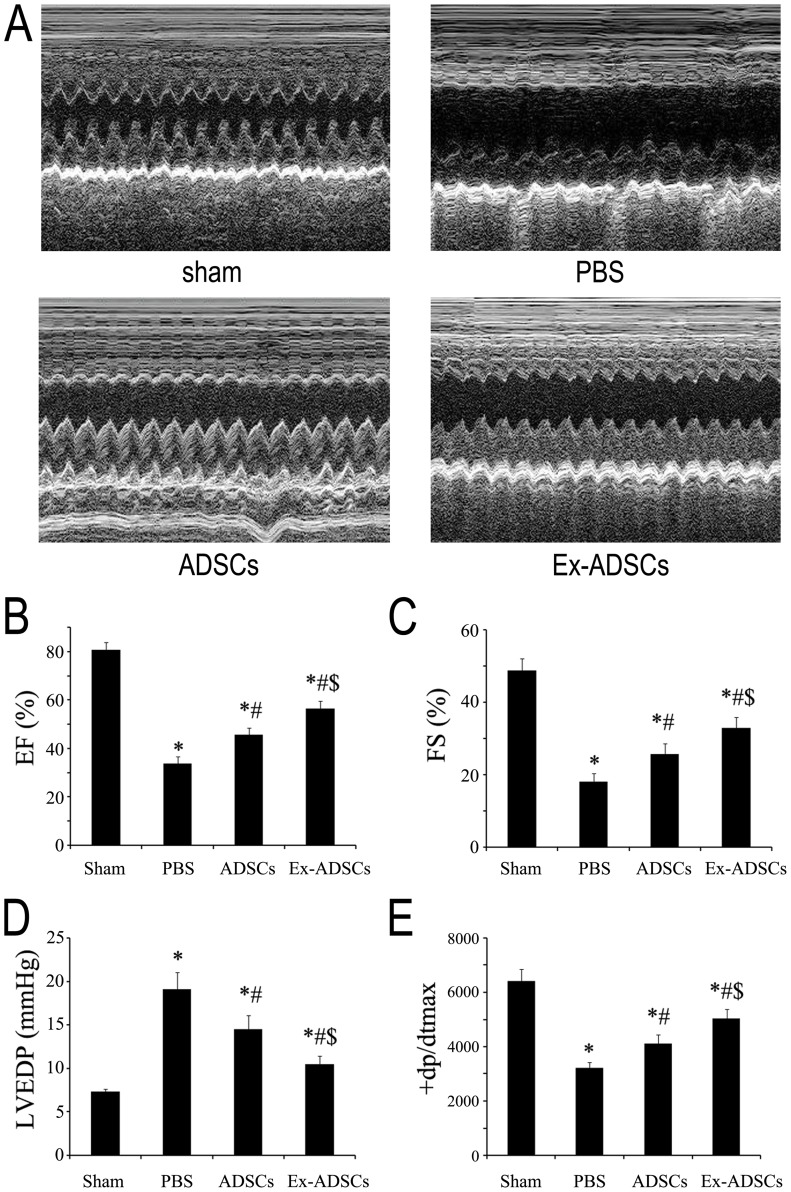
Echocardiographic Assessment of Cardiac Function (n = 16/group). (A) Representative M-mode echocardiograms in each group at day 28 after myocardial infarction. (B and C) Quantitative analysis of ejection fraction (B) and fractional shortening (C) by echocardiography. (D and E) Quantitative analysis of hemodynamic parameters LVEDP (D) and dP/dtmax (E). Statistical differences (*p*<0.05) are indicated from the sham (*), PBS (#), and ADSCs ($).

Left ventricular function of rats was assessed by echocardiogram at day 28 after transplantation (Fig. 4A). Left ventricular ejection fraction (LVEF) and left ventricular fractional shorting (LVFS) in the groups treated with Ex-ADSCs or ADSCs or PBS-alone had significantly declined compared with sham group (Fig. 4B, C, *p*<0.05). Injection of ADSCs and Ex-ADSCs significantly increased LVEF compared with PBS control (45.7±2.7%, 56.6±2.9%, *vs*. 33.7±3.0% respectively, *p*<0.05), with implantation of Ex-ADSCs highest (Fig. 4B). Similarly, the treatment using ADSCs or Ex-ADSCs injection improved LVFS markedly after 4 weeks compared with PBS control (25.7±3.0%, 32.9±2.9%, vs. 18.1±2.2% respectively, *p*<0.05). The best LV function was obtained for hearts with Ex-ADSCs injection (Fig. 4C).

In addition, invasive hemodynamic parameters showed decrease in left ventricular end-diastolic pressure (LVEDP) for both ADSCs and Ex-ADSCs groups compared to PBS group (14.5±1.6 mmHg in ADSCs group, 10.5±0.9 mmHg in Ex-ADSCs group vs. 19.1±2.0 mmHg in PBS group, *p*<0.05; Fig. 4D), the best effect was obtained in the Ex-ADSCs group. The maximum LV change in pressure over time (+dp/dtmax) was significantly improved by Ex-ADSCs and ADSCs groups compared with PBS group. The best effect was obtained in Ex-ADSCs group (*p*<0.05, Fig. 4E). These results suggested that pretreatment with Exendin-4 could significantly enhance the ADSC-based myocardial repair suffered from MI.

### Differentiation of ADSCs in infarcted myocardium

**Figure 5:**
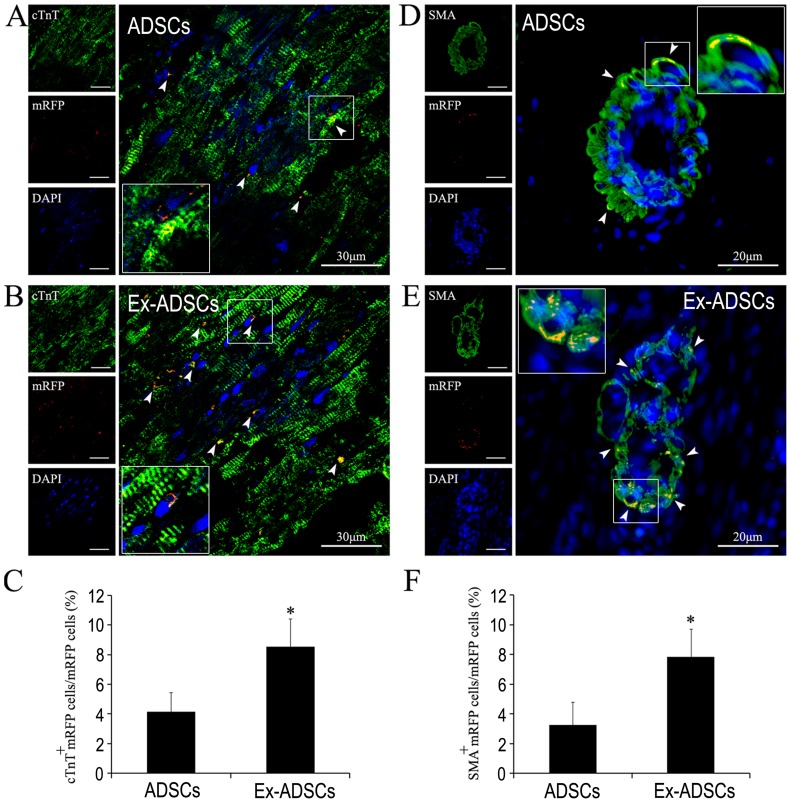
Confocal laser microscopic images of immunofluorescence analysis of differentiation of transplanted ADSCs in vivo (n = 12/group). (A and B) Representative images of differentiated cardiomyocytes-like cells using anti-cardiac troponin-I (green, cTnI) revealed significant augmentation of enhanced mRFP (red)/cTnI double positive cardiomyocyte-like cells (white arrow) in Ex-ADSCs group (B) compared with ADSCs group (A). (C) Quantitative analysis of the ratio of differentiated cardiomyocytes-like cells. (D and E) Representative images of differentiated vessel specific cells using anti-α-SMA (green) revealed significant enhancement of mRFP (red)/α-SMA double positive vessel-specific cells (white arrow) in Ex-ADSCs group (E) compared with ADSCs group(D). (F) Quantitative analysis of the ratio of differentiated vessel specific cells. *p<0.05. Inset shows the corresponding boxed area magnified.

Immunofluorescent analysis of cardiac- and blood vessel-specific proteins indicated that the hearts transplanted with Exendin-4-pretreated ADSCs contained an increased number of cells expressing cardiac marker (cTnT^+^/mRFP^+^, Fig. 5B) and vascular marker (SMA^+^/mRFP^+^, Fig. 5E) compared with that in the animals treated with ADSCs (Fig. 5A, D). The percentage of cTnT^+^/mRFP^+^ cells was much higher in the Ex-ADSCs group compared with that in the ADSCs group (8.52±1.91% *vs*. 4.15±1.32%, *P*<0.05, Fig. 5C). The percentage of SMA^+^/mRFP^+^ cells in the Ex-ADSCs group was significantly higher too than that in the ADSCs group (7.83±1.87% *vs*. 3.26±1.52%, *P*<0.05, Fig. 5F). The results suggested that Exendin-4 pretreatment could promote cardiac differentiation of engrafted ADSCs in ischemic myocardium.

### Apoptosis in the infarcted heart

**Figure 6:**
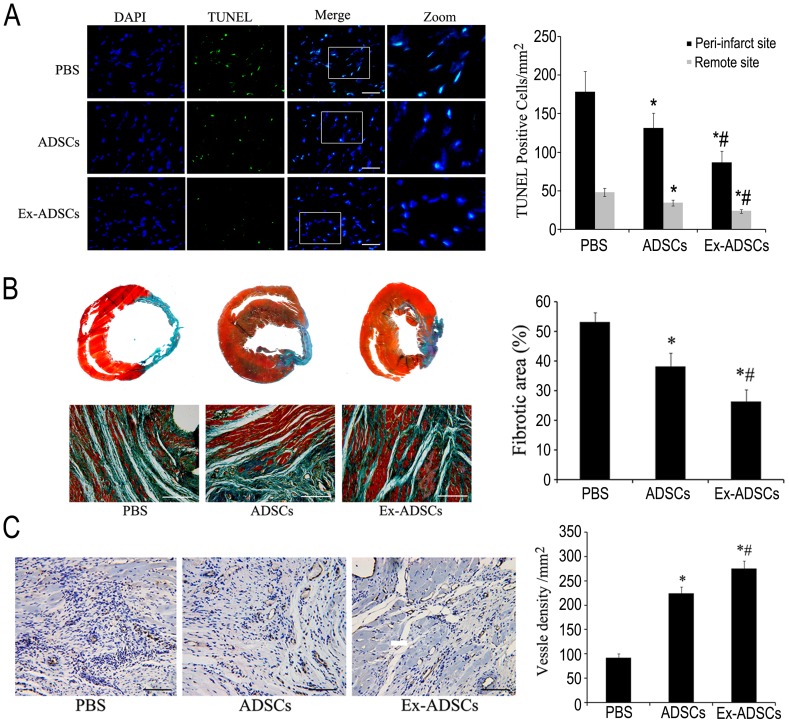
Transplantation of Exendin-4 pretreated ADSCs decrease apoptosis, fibrosis, and promote angiogenesis. (A) Representative TUNEL staining images and quantitative analysis in the peri-infarcted and remote zone of heart sections from each group (n = 4/group). Apoptotic nuclei were identified as TUNEL positive (green). Total nuclei were counterstained by DAPI (blue). Apoptotic cells nuclei were considered as apoptotic cardiomyocytes (white arrow). Scale bars = 50 µm. (B) Representative images and quantitative analysis of fibrotic area in different groups by Masson's trichrome staining (n = 12/group). Red represented viable myocardium, blue represented fibrosis. Scale bars = 200 µm. (C) Representative images and quantitative analysis of vessels intensity using anti-vWF antibody at the border zone of MI in each group by immunohistochemistry (n = 12/group). Scale bars = 100 µm. Statistical differences (*p*<0.05) are indicated from PBS (*) and ADSCs (#).

TUNEL staining demonstrated that apoptotic cells in peri-infarct site were more frequently observed in the PBS group and the ADSCs group compared with the Ex-ADSCs group (Fig. 6A). In the peri-infarcted zone, the percentage of apoptotic cells in groups ADSCs and Ex-ADSCs showed a decreasing trend compared with PBS group (131.5±19.3/mm^2^, 87.1±14.4/mm^2^ vs. 178.3±26.4/mm^2^, *p*<0.05. Likewise, in the remote non-infarcted myocardium, the percentage of apoptotic cells was 34.6±3.8/mm^2^ in ADSCs group, 23.8±2.4/mm^2^ in Ex-ADSCs group, and 48.2±5.3/mm^2^ in PBS group, the percentage of apoptotic cells in Ex-ADSCs group was significantly less among groups (*p*<0.05).

### Exendin-4 pretreated ADSCs reduces fibrosis after MI

Masson's trichrome staining on heart sections were performed at day 28 after infarction. Quantitative analyses demonstrated that there was a marked reduction in left ventricle fibrotic area in Ex-ADSCs group compared with the ADSCs or PBS group (Fig. 6B, *p*<0.05). These data suggest that transplantation of Exendin-4 pretreated ADSCs reduces fibrosis after MI.

### Exendin-4 Pretreatment enhances angiogenesis in ischemic myocardium

Microvessel density in the peri-infarct zone was evaluated based on vWF immunostaining 4 weeks after transplantation. The results showed that both the transplantation of ADSCs and Ex-ADSCs resulted in better microvessel densities than PBS (Fig. 6C). The microvessel density of the Ex-ADSCs group was the highest compared with ADSCs group and PBS group (*p*<0.05). The results suggested that Exendin-4 pretreatment could enhance the angiogenesis of ADSCs in ischemic myocardium.

## Discussion

The hostile microenvironment in the infarcted myocardium is a great challenge to the transplanted stem cells. It has been known that a high death rate of transplanted cells is an impediment of successful cell therapy [2], [3]. Several remedial strategies have been adopted to overcome the menace of anoikis (programmed cell death induced by loss of cell-matrix attachment) with unsatisfactory results [11], [13], [30]. Therefore, it is imperative to strengthen the implanted cells adhesion and viability to withstand the unfriendly niche in the ischemic heart. In this study, we found that Exendin-4 treatment could enhance ADSCs adhesion, survival and improve heart function in ischemic heart via reversing the impaired cellular adhesion induced by ROS.

Based on our observations, we demonstrated for the first time that Exendin-4 pretreatment decreased ROS production in ADSCs. Independent studies demonstrated that increased ROS in HUVECs and rat Goto-Kakizaki islets was decreased by treatment with GLP-1 and GLP-1R agonists [17], [18], which were analogous to our study. In addition, this effect was also observed in diabetic *db/db* mouse islets by treatment with an inhibitor of dipeptidyl peptidase IV that inhibits endogenous GLP-1 clearance [31]. In particular, a recent study showed that Exenatide suppressed ROS generation in the diabetic obese patient's peripheral blood mononuclear cells (MNC) [32]. On the other hand, ROS, generated in ischemic myocardium, could decrease the adhesion of mesenchymal stem cells (MSCs) and promote cell detachment and death through attenuation of integrin β1 and αV expression [9], [10], [33]. In this study, scavenging ROS by Exendin-4 pretreatment largely enhanced the adhesion of ADSCs, implicating that impaired adhesion of ADSCs are ROS dependent, which is in accordance with our previous report [10]. In parallel with Exendin-4 induced adhesion change, the mRNA levels of integrin molecules, such as β1 and αV, and the phosphorylation of focal adhesion kinase, such as FAK and Src, were down regulated in H_2_O_2_-treated ADSCs but the trends were partially attenuated by Exendin-4 pretreatment. In fact, previous study has confirmed that integrins-mediated adhesion were preferentially through β1 and αV [34]. In particular, integrin β1 has been identified as a distinctive pathway for MSCs migration and engraftment in myocardium [26]. Though our previous studies have shown that H_2_O_2_-induced integrins alteration inhibited FAK and Src complex expression in rat ADSCs, whether Exendin-4 has an influence on ADSCs adhesion through FAK-Src complex is still unknown. This study disclosed that H_2_O_2-_impaired adhesion related proteins of ADSCs such as FAK, Src, paxillin, vinculin, and talin, could be significantly reversed by Exendin-4 pretreatment. Moreover, we also observed that Exendin-4 pretreatment inhibited H_2_O_2_-induced caspase3 activity. Collectively, these results showed that Exendin-4 pretreatment facilitates ADSCs adhesion and survival through integrin-related focal adhesion proteins and caspase3 signaling. It should be noteworthy that less apoptosis could also exhibit better adhesion signaling.

Parallel observations were made when Exendin-4 pretreated ADSCs were transplanted in the infarcted rat heart. Based on serial invasive BLI imaging, the number of survived Ex-ADSCs was persistent higher than that of untreated ADSCs at the different time point. In addition, 4 weeks after MI, the majority of the imaging signals were no longer observed in ADSCs group, while there were still a few alive ADSCs in Ex-ADSCs group, indirectly indicating that Exendin-4 pretreatment improved cell survival in ischemic myocardium. Moreover, Ex-ADSCs significantly promoted cardiac function and viability at day 28. Histologically, transplantation of Ex-ADSCs significantly decreased infarct fibrosis, enhanced vessel density, and suppressed the myocardial apoptosis compared with transplantation of untreated ADSCs.

In stem cell based therapy, differentiation into the desired lineages (mainly cardiac and smooth muscle and endothelium) is the best option to definitively heal the scar. However, It is now accepted that the capability of ADSCs to improve left ventricular function is mainly through growth factor-mediated paracrine effects. The presence of cardiomyocytes within the ADSCs grafts appeared to be rare [35]. In our previous study, we showed that even delivered by injectable biomaterials, very few ADSCs could be detected to differentiate into cardiovascular lineages [10]. In this study, we showed that the percentage of cTnT^+^/mRFP^+^ and SMA^+^/mRFP^+^ cells in the Ex-ADSCs group were significantly higher than that in the ADSCs group(*p*<0.05, Fig. 5), indicating that Exendin-4 pretreatment could promote cardiac differentiation of engrafted ADSCs in ischemic myocardium. This is an intriguing finding since it offered a potential cure for the replacement of damaged myocardium. Reportedly, GLP-1 agonists could influence the stem cell differentiation, e.g. GLP-1 can mediate differentiation of human iPS cells and human ADSCs into insulin-secreting cells [36], [37]. However, the underlying molecular mechanism for the enhanced differentiation of ADSCs by Exendin-4 is unknown, which needs further investigation.

In conclusion, Exendin-4 pretreatment can enhance the resistance of ADSCs to ROS, facilitate cells adhesion and survival through integrin related adhesion proteins and caspase3 and improve ADSC-based myocardial repair. Transplantation of Exendin-4 pretreated ADSCs can be an innovative approach in cell therapy of myocardial infarction.

## Supporting Information

File S1 **Supporting materials. Figure S1, Characterization of ADSCs carrying fluc-mRFP reporter genes through lentiviral transduction.** (A): Flow cytometric analysis of ADSCs after stained by CD29, CD90, CD31, CD34 and CD45 antibodies. (B and C): The differentiation potential of ADSCs labeled with fluc-mRFP reporter. Scale bars = 100 µm. (D): Most of ADSCs expressed reporter after sorting. (E): *Ex vivo* bioluminescence signal intensity was positive proportional to cell numbers. **Methods S1, Isolation, characterization and lentiviral labeling of rat ADSCs.**
(DOC)
