# Supplementary material for: Exendin-4 Pretreated Adipose Derived Stem Cells Are Resistant to Oxidative Stress and Improve Cardiac Performance via Enhanced Adhesion in the Infarcted Heart
Source: PLoS One. 2014 Jun 10;9(6):e99756. doi: 10.1371/journal.pone.0099756 (PMC4051823; doi:10.1371/journal.pone.0099756)
Supplement: File S1 — Supporting materials. Figure S1, Characterization of ADSCs carrying fluc-mRFP reporter genes through lentiviral transduction. (A): Flow cytometric analysis of ADSCs after stained by CD29, CD90, CD31, CD34 and CD45 antibodies. (B and C): The differentiation potential of ADSCs labeled with fluc-mRFP reporter. Scale bars = 100 µm. (D): Most of ADSCs expressed reporter after sorting. (E): Ex vivo bioluminescence signal intensity was positive proportional to cell numbers. Methods S1, Isolation, characterization and lentiviral labeling of rat ADSCs. (DOC) [file pone.0099756.s001.doc]

**Supplementary Data**

**Manuscript title: Exendin-4 Pretreated Adipose derived Stem Cells are Resistant to Oxidative Stress and Improve Cardiac Performance via Enhanced Adhesion in the Infarcted Heart**

**Supplementary methods**

**Isolation, Expansion, and characterization of rat ADSCs**

Subcutaneous adipose tissue in inguinal groove was acquired under sterile conditions from autologous Sprague-Dawley rats and digested to obtain a stromal vascular fraction (SVF) as previously described with minor modifications . Briefly, adipose was rinsed with sterile phosphate-buffered saline (PBS), minced and enzymatically dissociated with composite of 0.1% collagnase I (sigma) and 0.05% trypsin (sigma) in serum-free alpha-modified Eagle’s medium (α-MEM, Gibco, USA) for 45-60 min at 37°C with gentle agitation. Then the enzyme was neutralized with an equal volume of α-MEM/10% fetal bovine serum (FBS, Gibco). The mixture was sequentially filtered through 75μm mesh filter to remove debris and centrifuged at 600g for 5min. the supernatant containing adipocytes and debris was discarded. The pelleted cells was resuspended in α-MEM supplemented with 10% FBS and plated onto 10cm culture plates. The dishes were incubated at 37°C, 5% CO_2_ incubator and the medium was replaced every 2-3 days to remove unattached cells. When cells reached 80-90% confluence within 3-5 days after the initial plating, adherent cells were detached with 0.25% trypsin/0.04% EDTA (v/v 1:1) and seeded at a ratio of 1:3. Cultures were passaged every 3-4 days and used for experimental procedures at passage 3 to 5.

**Flow Cytometry**

To confirm the immunophenotype of adherent cells, surface markers CD29, CD90, CD45, CD34, and CD31 were analyzed by fluorescence-activated cell sorting (FACS) (BD accrri C6). Briefly, cultured adherent cells isolated from adipose tissue were harvested and washed with cell staining buffer (Biolegend). Cell aliquots (1×10^6^ cells) were incubated for 30 min at 4°C with mouse monoclonal antibodies to rat CD34 (Santa Cruz), CD31(clone WM-51), CD45 (Biolgend), CD90 (Biolgend), and CD29 (Biolgend). For unlabeled antibodies, FITC-conjugated secondary antibodies were added. Then cells were incubated for another 30 min at 4°C. Labeled cells were washed in cell staining buffer twice, and then analyzed by FACS. Isotype-identical antibodies served as controls.

**Multi-differentiation assays**

To confirm multipotency of adherent cells, adipogenic and osteogenic differentiation were performed by Alizarin Red staining and Oil Red O staining, respectively. Briefly, for adipogenic differentiation, cells were seeded on the 6-well plate at a density of 1×10^5^ cells/cm^2^. After one day, the medium was replaced by adipogenic differentiation medium containing low-glucose Dulbeco’s modified Eagle’s medium (DMEM) supplemented with 10% FBS, 2 mM L-glutamine, 100 U/mL penicillin, 100 μg/mL streptomycin, 100 μM L-ascorbic acid (Sigma), 1 μM dexamethasone, 0.5 mM 1-methyl-3-isobutylxanthine, and 100 μM indomethacin. Then cells were cultivated for up to 21 days. For osteogenic differentiation, cells were seeded at a density of 1×10^5^/cm^2^. After reaching 100% confluence, cells were incubated in osteogenic differentiation medium (high-glucose DMEM supplemented with 10% FBS, 0.1 μM dexamethasone, 200 μM L-ascorbic acid, and 10 mM β-glycerol phosphate (Sigma) for 3 weeks. Osteogenic and adipogenic differentiation medium were changed every 3 days. Verification of adipogenic and Osteogenic differentiation were performed by Oil Red O staining and Alizarin Red staining respectively.

**Construction of lentiviral vectors carrying dual fusion reporter gene**

ADSCs were lentivirally transduced to express both firefly luciferase (fluc) and monomeric red fluorescent protein (mRFP) as described previously . Briefly, Plasmids vector containing fluc-mRFP fusion reporter gene, packaging system ps PAX2 and envelop plasmid pMD 2G were kindly provided by Dr. Sam. S. Gambhir (from Stanford University, Radiology Department). Three Vectors were cotransfected into 293T cells using Entranster^TM^–H reagent (Engreen Biosystem Co, Ltd.) according to manufacturer’s instruction. Lentivirus supernatant was concentrated in ultrafiltrate centrifuge tubes at 5000 g. Concentrated virus was titrated on 293T cells and frozen in -70°C for future use.

**Lentiviral labeling of Rat ADSCs**

For transduction, 1 × 10^3^ target cells/cm^2^ at passage 1 were plated in a 6-well cell culture plate. After 60-70% confluence, the cells were incubated with lentiviral vectors (at a multiplicity of infection of 15) in Opti-MEM (Gibco BRL) containing 8 μg/mL polybrene (Sigma) for 24h. After transduction, monomeric red fluorescence protein (mRFP) positive ADSCs were analyzed by FACScan (BD FACSVantage Diva). The 5% highest mRFP expressing cells were selected by FACS and expanded for several passages before usage.

**Reference**

1. Liu Z, Wang H, Wang Y, Lin Q, Yao A, et al. (2012) The influence of chitosan hydrogel on stem cell engraftment, survival and homing in the ischemic myocardial microenvironment. Biomaterials 33: 3093-3106.
2. Zhang X, Wang H, Ma X, Adila A, Wang B, et al. (2010) Preservation of the cardiac function in infarcted rat hearts by the transplantation of adipose-derived stem cells with injectable fibrin scaffolds. Exp Biol Med (Maywood) 235: 1505-1515.
3. Liu XB, Chen H, Chen HQ, Zhu MF, Hu XY, et al. (2012) Angiopoietin-1 preconditioning enhances survival and functional recovery of mesenchymal stem cell transplantation. J Zhejiang Univ Sci B 13: 616-623.
4. Cao F, Lin S, Xie X, Ray P, Patel M, et al. (2006) In vivo visualization of embryonic stem cell survival, proliferation, and migration after cardiac delivery. Circulation 113: 1005-1014.

**Supplementary Figure**

**
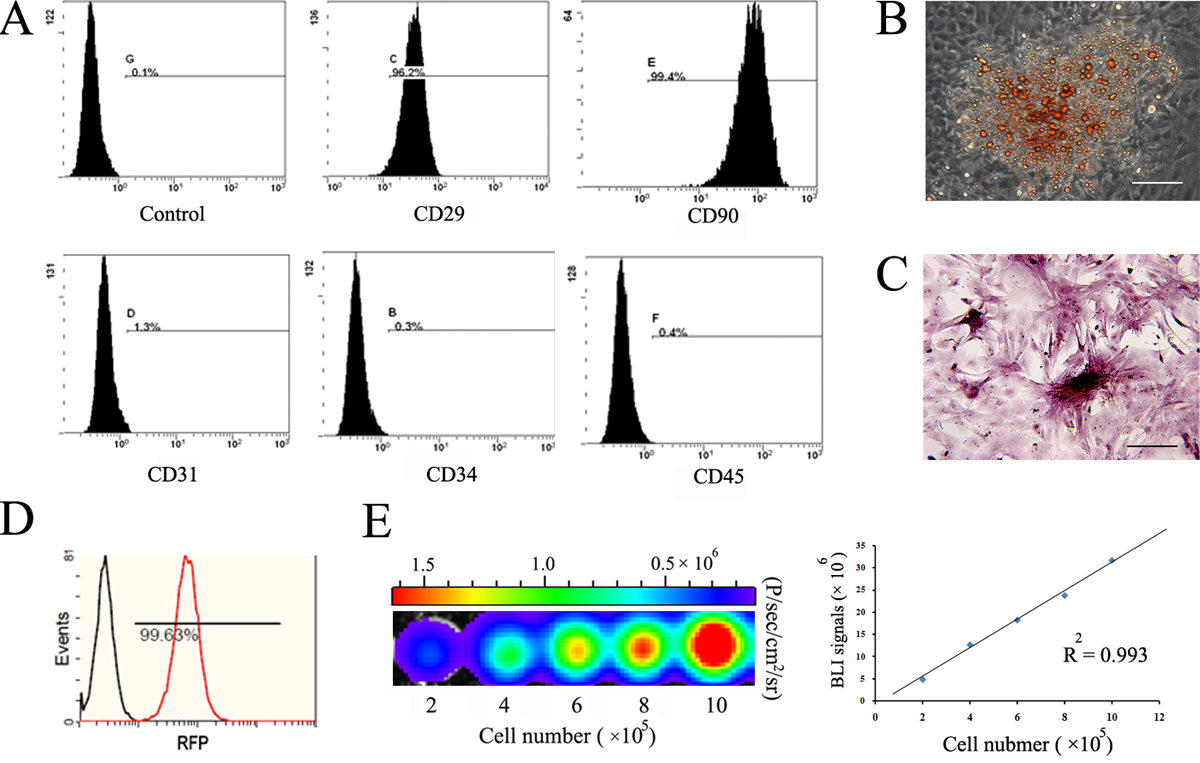
**

**Figure S1. Characterization of ADSCs carrying fluc-mRFP reporter genes through lentiviral transduction.** (A): Flow cytometric analysis of ADSCs after stained by CD29, CD90, CD31, CD34 and CD45 antibodies. (B and C): The differentiation potential of ADSCs labeled with fluc-mRFP reporter. Scale bars= 100μm. (D): Most of ADSCs expressed reporter after sorting. (E): *Ex vivo* bioluminescence signal intensity was positive proportional to cell numbers.
